# Cone-Beam Computed Tomography Evaluation of Mental Foramen Variations: A Preliminary Study

**DOI:** 10.1155/2015/124635

**Published:** 2015-11-02

**Authors:** Mahnaz Sheikhi, Mitra Karbasi Kheir, Ehsan Hekmatian

**Affiliations:** ^1^Torabinejad Research Center, Department of Oral and Maxillofacial Radiology, School of Dentistry, Isfahan University of Medical Sciences, Isfahan, Iran; ^2^Dental Students Research Center, Department of Oral and Maxillofacial Radiology, School of Dentistry, Isfahan University of Medical Sciences, Isfahan 8158974646, Iran

## Abstract

*Background*. Mental foramen is important in surgical operations of premolars because it transfers the mental nerves and vessels. This study evaluated the variations of mental foramen by cone-beam computed tomography among a selected Iranian population. *Materials and Methods*. A total number of 180 cone-beam computed tomography projections were analyzed in terms of shape, size, direction, and horizontal and vertical positions of mental foramen in the right and left sides. *Results*. The most common shape was oval, opening direction was posterior-superior, horizontal position was in line with second premolar, and vertical position was apical to the adjacent dental root. The mean of foremen diameter was 3.59 mm. *Conclusion*. In addition to the most common types of mental foramen, other variations exist, too. Hence, it reflects the significance of preoperative radiographic examinations, especially 3-dimensional images to prevent nerve damage.

## 1. Introduction

Mental foramen, an opening in the lateral surface of mandible, is important in surgical operations in the premolars because it transfers the mental nerves and vessels. Since foramen cannot be seen or touched clearly, identification of the actual clinical location of mental foramen can prevent nerve damage during surgeries and can contribute to administration of successful local anesthesia. It is seen to be oval or circular in shape. There are variations in the position, direction, size, and shape of foramen among different populations [1–4]. The only study on Iranian population was carried out by Haghanifar and Rokouei, which only determined the horizontal position of mental foramen in panoramic radiographs [5]. Cone-beam computed tomography (CBCT) provides 3D evaluation of mandible, and its measurement accuracy is superior to panoramic radiography. Recently, another study was conducted on a selected Iranian population which determined the horizontal position and direction of mental foramen [6]. However, a detailed study is required to cover all anatomical variations of mental foramen. So this study was aimed at evaluating the shape, size, direction, and horizontal and vertical positions of mental foramen by CBCT among a selected Iranian population.

## 2. Materials and Methods

A total of 180 CBCT images, taken from the patients for diagnostic purposes in the Department of Maxillofacial Radiology at Isfahan School of Dentistry from 2010 to 2015, were analyzed by two examiners (a maxillofacial resident and a radiologist) on an LG LED computer viewer (E2042C, Korea) using Sirona Galileos software. All CBCT images were taken by Sirona Orthophos, Galileos version 1.7 (Sirona, Germany), with a flat panel detector. The adjusted scan parameters were 85 Kvp and 10–42 mA depending on the size of patients. Exposure time was 14 seconds, effective exposure time was 2–6 seconds, and voxel size was 0.3*∗*0.3*∗*0.3 mm. The inclusion criteria included the patients older than 18 years whose skeletal growth was completed (partial or full edentulous and dentate patients). The exclusion criteria consisted of the patients below 18 years old and patients with pathologic lesions in mandible.

CBCT projections were analyzed in different planes (tangential, cross-sectional, and axial). Mental foramen was identified in cross-sectional and axial views. Then, the shape of mental foramen was determined from tangential projection (round and oval). In the same tangential section, in which the shape of foramen was determined, the foramen diameter was measured using the length measuring option on Galileos software (from the anterior border to the posterior border of foramen). Direction of mental foramen opening was recorded based on reconstructed 3D CBCT images according to the categories of Fabian: superior, posterosuperior, labial, mesial (anteriorly), and posterior [7].

**Figure 1:**
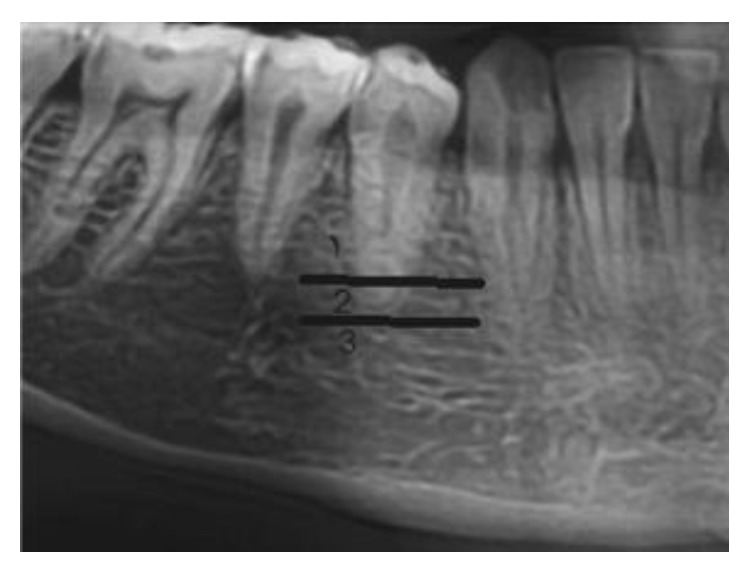
The vertical position of mental foramen image in relation to the dental root.

The vertical position of mental foramen image in relation to the dental root was recorded according to Fishel et al. and Green [8, 9] (Figure 1):

 Position 1: coronal to the apex of dental root.
 Position 2: at the apex of dental root.
 Position 3: apical to the apex of dental root.

**Figure 2:**
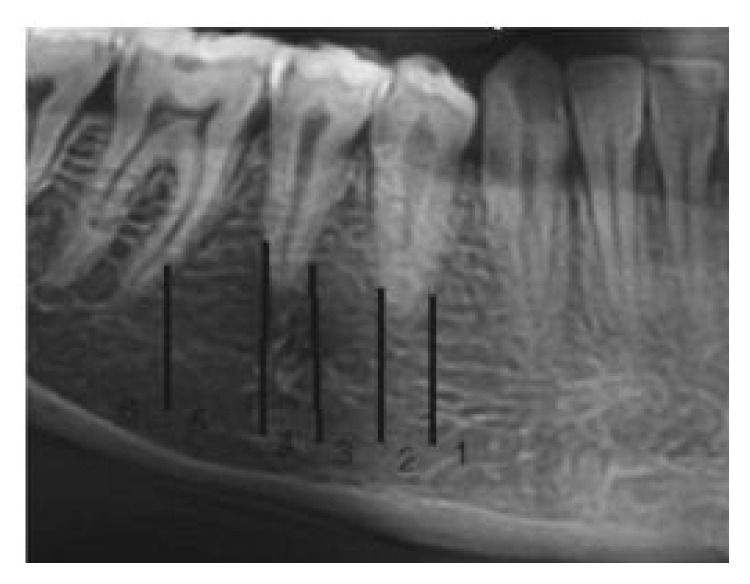
The horizontal position of mental foramen.

The horizontal position of mental foramen image was recorded according to Yosue et al. [10, 11] (Figure 2):

 Position 1: situated anterior to the first premolar.
 Position 2: in line with the first premolar.
 Position 3: between the first and second premolars.
 Position 4: in line with the second premolar.
 Position 5: between the second premolar and first molar.
 Position 6: in line with the first molar.

The data were analyzed by the statistical package for social sciences (SPSS) (version 22, SPSS Inc., Chicago, IL) and the level of significance was set at 0.05.

## 3. Results

From 180 CBCT images, 84 and 96 images belonged to men and women, respectively. The mean age of patients was 48 years (SD ± 13.9). There were 75% partial edentulous, 17.2% full edentulous, and 7.8% dentate in the right side and 75% partial edentulous, 16.7% full edentulous, and 8.3% dentate in the left side. Mental foramen was present in both sides in all of the images.

### 3.1. Mental Foramen Shape

The frequency of mental foramen shape is as follows. In the right side, 69.4% of images were oval and 30.6% were round. In the left side, 67.8% of images were oval and 32.2% were round. 82.8% of mental foramen shape in images was round or oval in both sides (22.8% round and 60% oval in the both right and left sides). Kappa Coefficient was 0.6 (*P* value < 0.001) and *P* value of McNamara test was 0.72.

For males, the frequency of round mental foramen in the right side was 36.9% and in the left side was 29.8% and the frequency of oval mental foramen in the right side was 63.1% and in the left side was 70.2%. For females, the frequency of round mental foramen in the right side was 28.1% and in the left side was 31.3% and the frequency of oval mental foramen in the right side was 71.9% and in the left side was 68.8%. The results of chi-square test did not show a statistically significant difference between mental foramen shape and gender in the right and left sides (the right side *P* value = 0.21 and the left side *P* value = 0.83).

The mean age of round mental foramen in the right side was 45 (SD ± 17) years and in the left side was 46 (SD ± 15) years. The mean age of oval mental foramen in the right side was 50 (SD ± 11) years and in the left side was 49 (SD ± 13) years. The findings of *t*-test showed a statistically significant difference between the mean age of samples and foramen shape in the right side (*P* value = 0.04) but did not show a statistically significant difference between the mean age of samples and foramen shape in the left side (*P* value = 0.27).

### 3.2. Mental Foramen Size

The mean diameter of mental foramen was 3.59 mm (SD ± 2.74) in the right side and 3.59 mm (SD ± 1.17) in the left side.

### 3.3. The Opening Direction of Mental Foramen

The frequencies of mental foramen opening direction are presented in Table 1. The agreement indices of foramen opening direction between the right and left sides were 20% superior, 53.9% posterosuperior, 1.7% labial, 1.7% mesial, and 1.7% posterior, with kappa coefficient of 0.59 (*P* value < 0.001), indicating a relative agreement between the right and left sides in terms of opening direction.

The frequencies of mental foramen opening directions for males and females in the right and left sides are presented in Table 2. The results of chi-square test did not show significant differences between mental foramen opening direction and gender (right side *P* value = 0.28 and left side *P* value = 0.15).

Table 2 shows the mean age of samples in different opening directions of mental foramen in the right and left sides. The findings of one-way ANOVA test showed a significant difference between different directions of mental foramen opening and mean age in the right side (*P* value = 0.02) but did not show a significant difference in the left side (*P* value = 0.09). Moreover, the findings of Tukey HSD showed a significant difference between the posterior and superior directions in the right side (*P* value = 0.04) and the sample's mean age in the superior direction of mental foramen opening was higher than that of posterior direction.

### 3.4. Vertical Location of Mental Foramen

The frequency of vertical location of mental foramen is presented in Table 1. Analysis was done between dentate cases. There was a low agreement in vertical location of mental foramen in the right and left sides. Kappa coefficient was reported to be 0.26 (*P* value = 0.03).

The results of chi-square test did not show statistically significant differences between different vertical locations of mental foramen and gender (right side *P* value = 0.38 and left side *P* value = 0.16).

The results of one-way ANOVA for dentate cases showed no significant difference between the vertical location of foramen and mean age in the right (*P* value = 0.09) and left (*P* value = 0.25) sides.

### 3.5. Horizontal Location of Mental Foramen

The location of mental foramen in the horizontal plane is shown in Table 1. The results of analysis for dentate cases indicated that 22.7% of samples were located between the first premolar and second premolar, 18.2% were in line with second premolar, and 6.8% were between the second premolar and first molar in both sides, so there was not any agreement between the horizontal locations of mental foramen in the right and left sides. Kappa coefficient was 0.19 (*P* value = 0.07).

The findings of chi-square test did not show statistically significant differences between different horizontal locations of mental foramen and gender (right side *P* value = 0.79 and left side *P* value = 0.45). The results of one-way ANOVA for dentate cases showed no significant difference between the horizontal locations of mental foramen and mean age in the right (*P* value = 0.80) and left (*P* value = 0.55) sides.

## 4. Discussion

Mental foramen is an important landmark of mandible. Determination of anatomic variations of mental foramen has been the subject of many studies. These variations provide the ground to avoid surgical nerve damage and to gain successful anesthesia.

### 4.1. Mental Foramen Shape

Many studies have reported two types of mental foramen shape, oval and round; they have demonstrated differences between races. The majority of Indians were reported to have round mental foramen while Malawians have mostly oval one. Round and oval shapes were reported to gain approximately equal percentage in Tanzanian and Zimbabwean populations [7, 12]. In our study, oval shape was twice greater than round shape, which is in accordance with Gershenson et al. [13]. The shape of mental foramen was the same in both sides in many of our cases (82.8%). There were no statistically significant differences between foramen shapes and sex in the right and left sides. Our findings showed that mental foramen shape did not change with age increase.

### 4.2. Mental Foramen Size

Mental foramen size in our study was similar to those reported by Neiva et al. among Caucasians and Yosue et al. [10, 11, 14].

### 4.3. The Opening Direction of Mental Foramen

The opening direction of mental foramen has been evaluated in some races [7, 15]. The nearest race to Iranian population is Caucasian which has been studied by Kieser et al. As they concluded, the most common type of direction in Caucasian population was posterior [16]. Our study was different than theirs, because superior-posterior was the most common direction. However, our findings were in accordance with the results of the study by Khojastepour et al. in which they reported posterior direction as the least common type although they used different classification for direction [6]. Our study can support the theory that mental foramen direction shows changes with age increase. During the development of mandible, the direction alters from the forward to upward and backward direction [12]. Our study confirmed this as the study population was chosen from the patients older than 18 years, whose skeletal growth had been completed consequently and their most common direction was superior-posterior. Besides, the mean age in the posterior type was lower than that of the others. This study showed an agreement in the opening direction of mental foramen in the right and left sides, and foramen opening direction was reported not to be gender-dependent.

### 4.4. Vertical and Horizontal Locations of Mental Foramen

The literature suggests that the location of mental foramen shows differences according to race, which was confirmed by our study. Besides, this study showed foramen location was not gender- and age-dependent and there was not any agreement in the horizontal and vertical locations of mental foramen in the right and left sides. This is in agreement with Al Jasser and Nwoku's study, which showed the location of mental foramen was not gender-dependent [2]. The most common location of foramen in vertical plane in our study was apical to the apex, which was similar to the results of other studies [8]. In our study, the most common horizontal locations were in line with the second premolar and between the first premolar and second premolar. Similarly, Haghanifar and Rokouei showed horizontal positions 3 and 4 were the most common types [5]. Khojastepour et al. divided horizontal position into three types which was different from our classification; however, the results were similar [6]. It seems horizontal location of mental foramen is race-dependent. According to Al-Juboori et al, horizontal location of mental foramen was more posterior in blacks than in whites and the most common horizontal locations in Caucasian population were Positions 3 and 4 [12]. However, Positions 1, 2, 5, and 6 have been reported to have low incidence in other studies [2, 4, 7, 15].

Determining the position of mental foramen from the adjacent teeth has limitations in many patients, as many of the implant applicants are partially or fully edentulous and have lost their molars and premolars a long time ago. Thus, further studies are recommended to analyze the invariable landmarks like the inferior border of mandible to locate mental foramen.

## 5. Conclusion

According to the present study, the most common variation of mental foramen in Iranian population was oval, superior-posterior opening, apical to the apex, and in line with second premolar. However, other types of mental foramen variables exist, reflecting the significance of preoperative radiographic examinations, especially 3D images (CBCT). These findings can help in safe implant surgery and administration of successful local anesthesia.

## Figures and Tables

**Table 1:** The frequency (%) direction of opening, vertical, and horizontal locations of mental foramen.

Variables		Foramen in right side	Foramen in left side
The opening direction of mental foramen	Superior	27.2	30.6
	Superior-posterior	64.4	62.2
	Labial	5	2.2
	Mesial	1.7	3.3
	Posterior	1.7	1.7

Vertical location of mental foramen	Coronal to the apex	1.7	2.2
	At the apex	6.1	7.8
	Apical to the apex	24.4	31.1
	No tooth	67.8	58.9

Horizontal location of mental foramen	Anterior to PM1	0	0
	PM1	1.1	0.6
	PM1-PM2	11.1	17.2
	PM2	14.4	17.2
	PM2-M1	5.6	6.1
	M1	0	0
	No tooth	67.8	58.9

**Table 2:** The frequency (%) of the opening direction of mental foramen for gender and mean age in the right and left sides.

Variables	Superior	Superior-posterior	Labial	Mesial	Posterior	*P* value
R						
Male	23.8%	70.2%	3.6%	0%	2.4%	0.28
Female	30.2%	59.4%	6.3%	3.1%	1%	
L						
Male	23.8%	70.2%	2.4%	1.2%	2.4%	0.15
Female	36.5%	55.2%	2.1%	5.2%	1%	
R						
Mean age	53.14 (SD ± 13.8)	47.22 (SD ± 13)	47.33 (SD ± 18.7)	48 (SD ± 9.53)	30.33 (SD ± 17)	0.02
L						
Mean age	50.73 (SD ± 14.5)	47.63 (SD = 13.5)	53 (SD ± 5.7)	52.50 (SD ± 8.8)	30.33 (SD ± 17.2)	0.09
